# Topical Probiotic Formulation Promotes Rapid Healing in Dog Keratinocyte Cells: A Promising Approach for Wound Management

**DOI:** 10.3390/ijms241512360

**Published:** 2023-08-02

**Authors:** Manon Barthe, Lionel Gillot, Laurie Perdigon, Aline Jacobs, Gregory Schoonbroodt, Paul Mauhin, Emna Bouhajja, Hanan Osman-Ponchet

**Affiliations:** 1 PKDERM Laboratories, 06130 Grasse, France; manon.barthe@pkderm.com (M.B.); laurie.perdigon@pkderm.com (L.P.); 2 Probiotic Group Luxembourg S.A., Research and Development Department, 9944 Weiswampach, Luxembourg; lionel@probiotic-group.com (L.G.); aline@probiotic-group.com (A.J.); gregory@probiotic-group.com (G.S.); paul@probiotic-group.com (P.M.)

**Keywords:** probiotic, wound healing, dog keratinocytes, cell migration, topical formulation

## Abstract

The use of probiotics has gained increasing attention as a strategy for wound healing to decrease microbial resistance to disinfectants and antibiotics. This study aimed to investigate the potential of a non-medicinal topical cocktail of probiotic bacteria (CPB) in promoting wound healing in dogs using in vitro scratch assay. Canine Progenitors Epidermal Keratinocytes (CPEK) were exposed to a prototype product formulated with CPB (PPP), non-formulated CPB, and the vehicle. The viability of CPB and CPEK cells was first evaluated in the co-culture model. Then, wound closure was analyzed over time. The CPB required a minimum concentration of 75 CFU/mL for better viability with CPEK. While the CPEK preserved 100% of their viability when PPP was diluted to up to 75,000 CFU/mL. At higher concentrations, the viability of CPEK was reduced by the concomitant effect of the non-formulated CPB and the vehicle. The formulated and non-formulated CPB and the vehicle seem to lead to a dose-dependent increase in cell migration compared to the control. Importantly, at the concentration of 750,000 CFU/mL, the PPP showed a 20% increase in wound closure. Taken together, our findings suggest the potential beneficial effects of the probiotic-based topical cocktail (PPP) on wound healing. However, to confirm and validate these effects, further experiments are necessary to provide more robust evidence and allow us to confidently establish the potential beneficial effects of the probiotic bacteria (CPB) in promoting wound healing.

## 1. Introduction

Dogs are a favorite household pet. Their number was estimated to be 90 million in 88 million pet-owning households in Europe (European Pet Food Federation, 2021). They are present in 65.1 million households in the U.S. (© Statista 2023). Regardless of their lifestyle, dogs are prone to accidents and injuries which disrupt the skin tissue continuity giving place to wounds. Independently of their severity, wounds require the immediate attention of the owner and the veterinarian. In all vertebrates, the skin is a barrier that plays a crucial role in maintaining homeostasis and preventing the invasion of pathogens and chemical and physical insults. The damaged skin is susceptible to microbial invasion, leading to infected wounds. Instantly, the skin activates the wound healing as a self-regeneration after damage to restore the protective barrier. The process of healing is complex and involves inter-cellular interactions, growth factors, and cytokines. It encompasses overlapping phases starting with hemostasis, then inflammation, reparation, and maturation [1,2,3]. In veterinary practices, wound management is carried out with conventional antiseptic solutions (e.g., sterile saline, diluted chlorhexidine, or diluted betadine) and topical antimicrobial agents [1,2]. These solutions eliminate beneficial bacteria and pathogens, disrupting the balance of the skin microbiota [4]. In addition, the activation of immune protection leads to the perturbance of the microbiota balance on the skin. To address this challenge, the use of probiotics has emerged as a promising approach to regulate inflammation and microbiota balance to potentially enhance the healing process [5,6,7,8]. Probiotics have shown promising results in promoting wound healing in vivo animal models using for example *L. plantarum*, kefir, *L. fermentum*, and *S. cerevisiae* to treat burn or surgical wounds [9]. In a meta-analysis by Tsiouris et al. in 2017 [10], the sterile kefir extract and bacteria probiotic therapies (70% kefir gel, *L. brevis*, *L. fermentum*, *L. plantarum*, *L. reuteri*) were found to be accelerators of wound contraction. For improved efficacy, probiotics could be combined with other techniques, e.g., nanotechnology-based techniques [11].

In this study, our primary objective was to assess the potential of a non-medicinal topical cocktail of probiotic bacteria (CPB) in promoting wound healing in dog keratinocytes to provide support for the development of a probiotic-based topical cocktail intended for canine care purposes. Specifically, we focused on evaluating the influence of probiotics on cell migration in vitro using dog keratinocytes and scratch wound healing assay. The investigation involved multiple aspects. Firstly, we conducted viability tests on both probiotics and keratinocytes in co-culture to ensure their compatibility and assess their overall health. Subsequently, we investigated the wound healing process using the scratch test, allowing us to observe and analyze the effects of the CPB on cell migration and wound closure. Additionally, we examined the immune stimulation of keratinocytes through the quantification of cytokine expression levels. Through these comprehensive evaluations, we aimed to gain insights into the potential wound healing properties of the CPB and provide a scientific basis for the development of the PPP for use in canine care.

## 2. Results

### 2.1. Viability of Probiotic Bacteria in Culture Conditions

**Figure 1:**
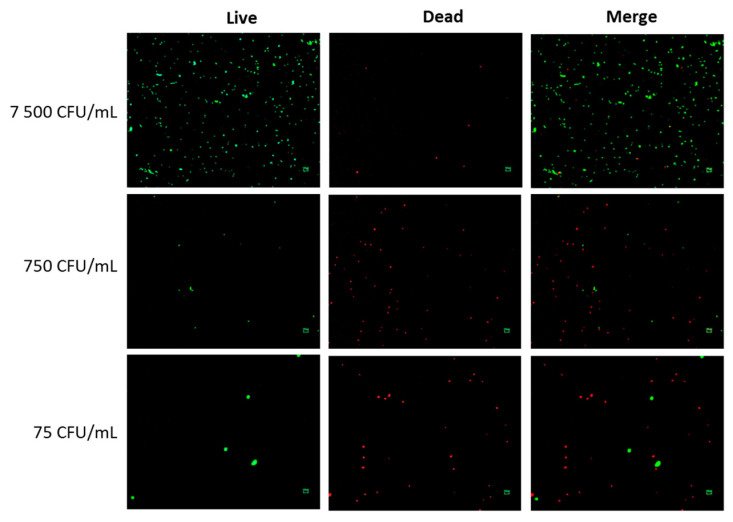
Live/dead bacterial viability assay of non-formulated cocktail probiotic bacteria. Canine Progenitors Epidermal Keratinocytes (CPEK) cultured in a 96-well plate were treated with non-formulated probiotic bacteria at 75, 750, or 7500 CFU/mL for 24 h. The bacteria were then stained with the BacLight™ double staining kit. The live and dead bacteria exhibited green and red fluorescence, respectively. The scale bar represents 100 µm.

The CPEK were exposed to non-formulated CPB at three different concentrations: 75, 750, and 7500 CFU/mL for a duration of 24 h. When exposed to the concentration of 7500 CFU/mL, there were very few instances of dead bacteria, accounting for only 2% (Figure 1). However, at concentrations of 750 CFU/mL and 75 CFU/mL, a significantly higher number of dead bacteria were observed, representing 81% and 84%, respectively. Moreover, the concentration of 7500 CFU/mL exhibited a significantly greater number of viable bacteria compared to the lowest concentration of 75 CFU/mL (279 versus 5). This finding supports the inverse relationship between the number of viable bacteria and the number of dead bacteria, as higher concentrations result in fewer instances of dead bacteria.

### 2.2. Effect of the Cocktail of Probiotic Bacteria on the Viability of CPEK Cells

**Figure 2:**
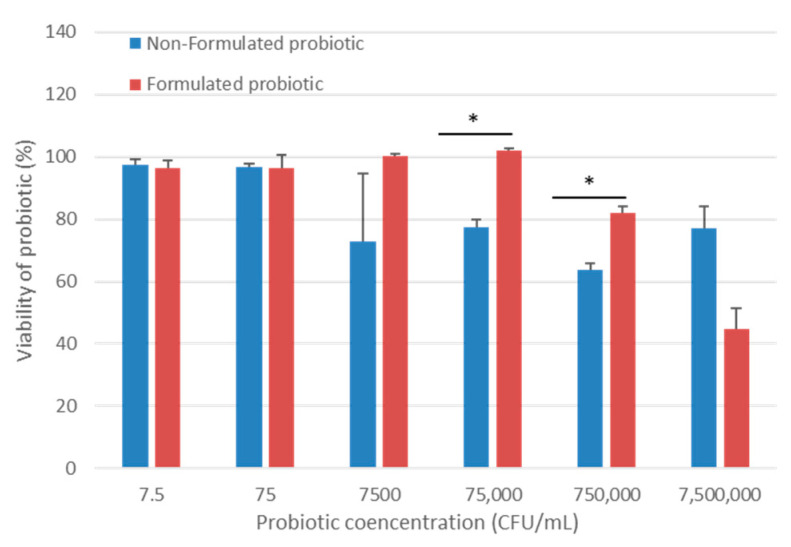
MTT cell viability assay. Canine Progenitors Epidermal Keratinocytes (CPEK) were untreated or treated with formulated or non-formulated probiotic bacteria at various concentrations for 24 h, and cell viability was determined using a colorimetric MTT assay. The results revealed that both the formulated and non-formulated probiotic bacteria were well tolerated by CPEK cells, except at very high concentrations. Here, the control (untreated) group is referred to as 100% viable cells. Data are presented as the mean ± standard deviation of one experiment conducted in duplicate (N = 2). * indicates (*p* < 0.05).

The CPEK were exposed to various concentrations of both formulated and non-formulated cocktail of probiotic bacteria, ranging from 7.5 to 7,500,000 CFU/mL, while untreated cells were used as a control. Following a 24 h period of co-culture with non-formulated CPB, a decrease in cell viability was observed (e.g., viability of 63.6% in the co-culture with 750,000 CFU/mL) only at concentrations above 75 CFU/mL of CPB (Figure 2). However, at lower concentrations of 75 CFU/mL and 750 CFU/mL of CPB, minimal to no cytotoxicity was observed. However, 100% of the viability of cells was maintained in the co-culture with up to 75,000 CFU/mL of formulated CPB. The highest concentration of the latter induced a higher decrease in the viability of cells than the non-formulated CPB, 44.8% and 77.2%, respectively. Taken together, these results indicated that the vehicle of formulated probiotic bacteria protected the CPEK from CPB toxicity when diluted to up to 75,000 CFU/mL This protection decreased with higher concentrations and could induce 32.4% of toxicity to CPEK in addition to the 44.8% of non-formulated CPB.

### 2.3. Effect of the Cocktail of Probiotic Bacteria on Cytokine Expression in CPEK Cells

**Figure 3:**
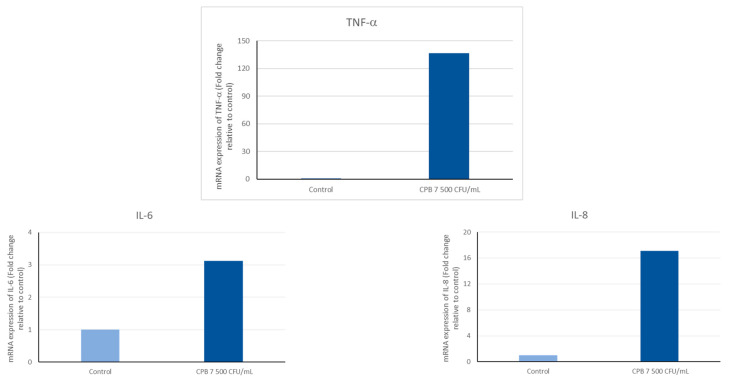
Effect of the cocktail of probiotic bacteria on mRNA expression of cytokine markers in CPEK cells. Canine Progenitors Epidermal Keratinocytes (CPEK) were exposed to non-formulated probiotic bacteria at 7500 CFU/mL for 24 h. mRNA expression of IL-6, IL-8, and TNF-α was measured using quantitative RT-PCR.

The CPEK were exposed to non-formulated CPB at a concentration of 7500 CFU/mL for a duration of 24 h. Following the treatment, the mRNA expression levels of the inflammatory cytokine markers, namely IL-8, IL-6, and TNF-α, were assessed using quantitative real-time RT-PCR. The findings depicted in Figure 3 demonstrate that the exposure to CPB at 7500 CFU/mL resulted in a substantial upregulation of IL-6, IL-8, and TNF-*α* mRNA expression, with fold increases of 3, 17, and 136, respectively. Moreover, an increase in IL-8 mRNA expression was observed even at the lowest concentration of 75 CFU/mL.

### 2.4. Effect of the Cocktail of Probiotic Bacteria on Wound Healing

**Figure 4:**
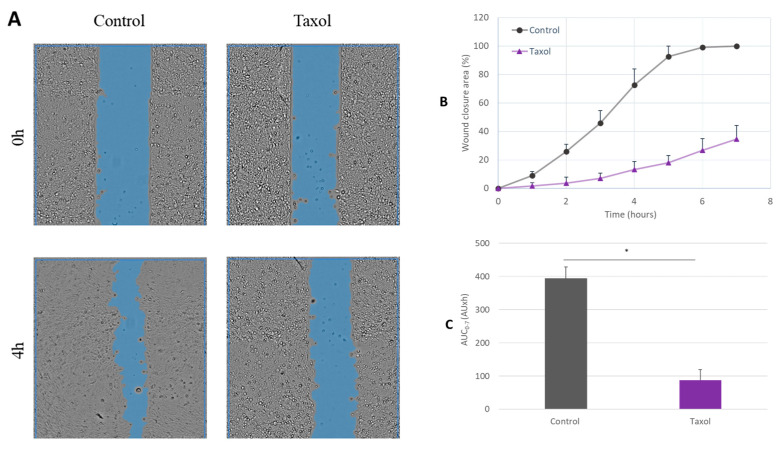
Scratch wound healing assay—the effect of taxol (positive control) on wound healing. Canine Progenitors Epidermal Keratinocytes (CPEK) were seeded into Ibidi^®^ inserts in a 24-well plate and allowed to adhere overnight. Precise and reproducible wounds were created when removing the inserts. Phase contrast images were acquired every hour using CytoSmart Omni live-cell analysis system. (**A**) Representative phase contrast images of CPEK cells at 0 h and 4 h post-wounding. Wound areas are highlighted in blue. The integrated image analysis algorithm automatically selects the cell-free areas. (**B**) Progression of scratch wound closure monitored over time in CPEK cells. The wound closure area is expressed as percentage, both in the absence and presence of 100 nM taxol. The scratch area at the 0 h time point was set to 100. (**C**) The area under the curve (AUC) for the time period of 0 to 7 h was calculated from the line graph. The measurement data are presented as the mean ± SD of two separate experiments conducted in duplicate (N = 4). * indicates (*p* < 0.05) compared to the control group.

The CPEK were exposed to different treatments, including formulated and non-formulated CPB, as well as the vehicle used in the formulation of probiotics. The experimental setup was validated by comparing the wound closure in the positive control treated with taxol and the untreated control (Figure 4). In fact, the control of untreated cells had an AUC (0 h–7 h) of 395 AU·h, whereas cells treated with 100 nM of taxol exhibited an AUC (0 h–7 h) of 88 AU·h. These findings indicate a significant reduction of cell migration by 78% with taxol treatment (*p*-value < 0.05).

**Figure 5:**
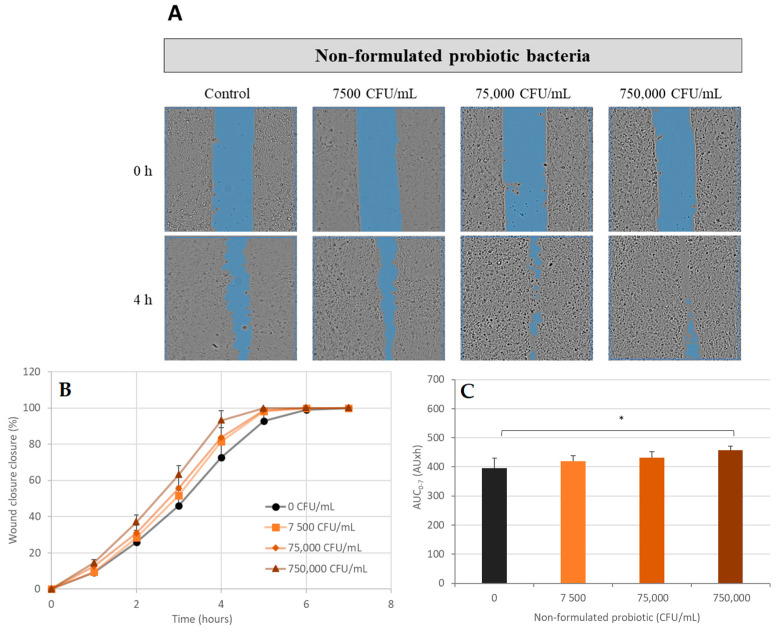
Scratch wound healing assay—the effect of non-formulated probiotic bacteria on wound healing. Canine Progenitors Epidermal Keratinocytes (CPEK) were exposed to non-formulated probiotic bacteria at 7500, 75,000, and 750,000 CFU/mL. (**A**) Representative phase contrast images of CPEK cells at 0 h and 4 h post-wounding. Wound areas are highlighted in blue. The integrated image analysis algorithm automatically selects the scratch areas. (**B**) Progression of scratch wound closure monitored over time in CPEK cells. The wound closure area is expressed as percentage, both in the absence and presence of non-formulated probiotic bacteria. (**C**) The area under the curve (AUC) for the time period of 0 to 7 h was calculated from the line graph. The measurement data are presented as the mean ± SD of two separate experiments conducted in duplicate (N = 4). * indicates (*p* < 0.05) compared to the control group.

In the presence of non-formulated CPB, CPEK had a dose-dependent increase in their migration (Figure 5A). In this condition, the AUC (0 h–7 h) of wound closure percentages were 419 AU·h at 7500 CFU/mL, 432 AU·h at 75,000 CFU/mL, and 458 AU*h at 750,000 CFU/mL (Figure 4C and Figure 5B). These findings showed that the treatment of CPEK cells with non-formulated cocktail of probiotic bacteria resulted in a respective enhancement of cell migration by 6%, 9%, and 16% (*p*-value < 0.05).

**Figure 6:**
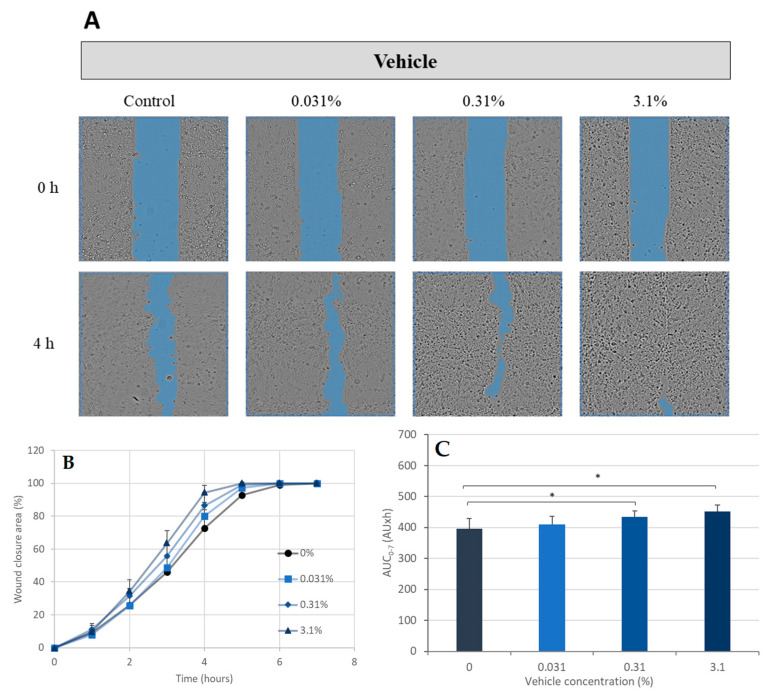
Scratch wound healing assay—the effect of vehicle on wound healing. Canine Progenitors Epidermal Keratinocytes (CPEK) were exposed to the vehicle used in the formulation of the cocktail of probiotic bacteria at 0.031%, 0.31%, and 3.1%. (**A**) Representative phase contrast images of CPEK cells at 0 h and 4 h post-wounding. Wound areas are highlighted in blue. (**B**) Progression of scratch wound closure monitored over time in CPEK cells. The scratch area at the 0 h time point was set to 100. (**C**) The area under the curve (AUC) for the time period of 0 to 7 h was calculated from the line graph. The measurement data are presented as the mean ± SD of two separate experiments conducted in duplicate (N = 4). * indicates (*p* < 0.05) compared to the control group.

In addition to the CPB increase in CPEK migration, the vehicle used in the formulation of CPB had a dose-dependent effect on the cells as well (Figure 6A). The AUC (0 h–7 h) values were 410 AU·h, 434 AU·h, and 452 AU·h for the tested concentrations of the vehicle 0.031%, 0.31%, and 3.1%, respectively (Figure 6B,C). This increase was equivalent to 4%, 10% (*p*-value < 0.05), and 14% (*p*-value < 0.05), respectively.

**Figure 7:**
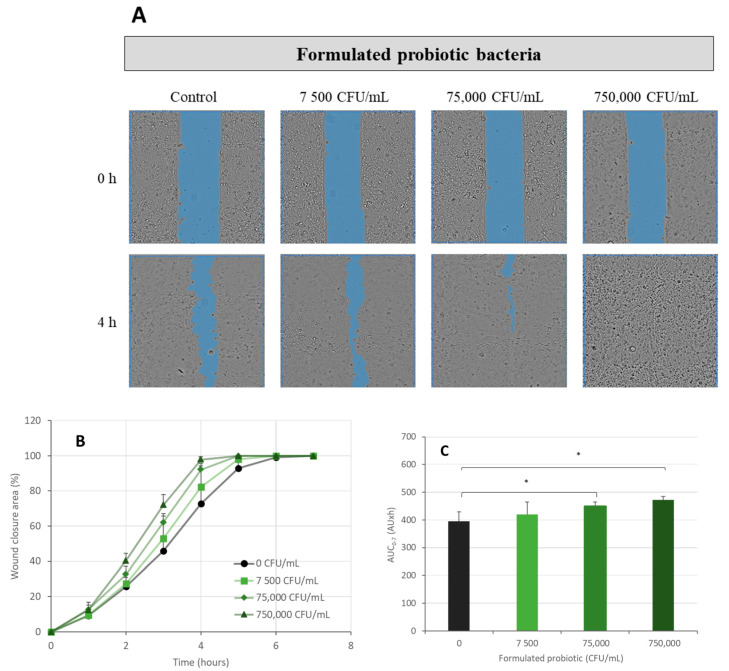
Scratch wound healing assay—the effect of formulated probiotic bacteria on wound healing. Canine Progenitors Epidermal Keratinocytes (CPEK) were exposed to formulated probiotic bacteria at 7500, 75,000, and 750,000 CFU/mL. (**A**) Representative phase contrast images of CPEK cells at 0 h and 4 h post-wounding. Wound areas are highlighted in blue. (**B**) Progression of scratch wound closure monitored over time in CPEK cells. (**C**) The area under the curve (AUC) for the time period of 0 to 7 h was calculated from the line graph. The measurement data are presented as the mean ± SD of two separate experiments conducted in duplicate (N = 4). * indicates (*p* < 0.05) compared to the control group.

The formulated CPB was a combination of non-formulated probiotics and the vehicle. It was tested at the same concentration as the non-formulated CPB. It showed a higher increase in wound closure speed over time (Figure 7). The AUC (0 h–7 h) were 420 AU·h, 449 AU·h, and 473 AU·h for the concentrations 7500 CFU/mL, 75,000 CFU/mL, and 750,000 CFU/mL, respectively. These findings indicated that the treatment of CPEK cells with the formulated probiotic bacteria resulted in an enhancement of cell migration by 6%, 14% (*p*-value < 0.05), and 20% (*p*-value < 0.05), for the tested concentrations, respectively.

**Figure 8:**
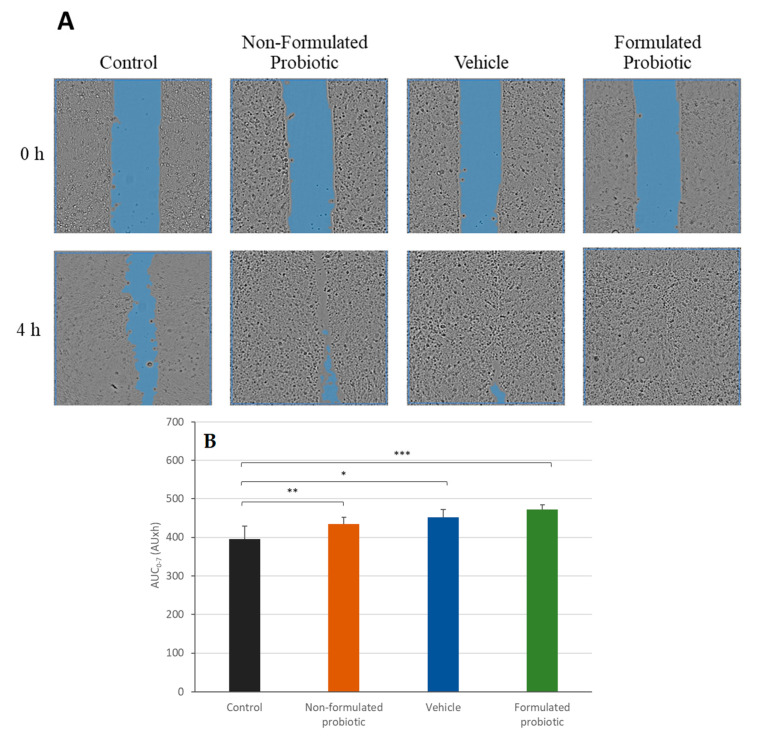
Scratch wound healing assay. Comparison of formulated and non-formulated probiotic bacteria on wound healing. Canine Progenitors Epidermal Keratinocytes (CPEK) exposed to formulated or non-formulated probiotic bacteria at 750,000 CFU/mL, or with the vehicle alone at 3.1%. (**A**) Representative phase contrast images of CPEK cells at 0 h and 4 h post-wounding. Wound areas are highlighted in blue. (**B**) Area under the curve (AUC) for the time period of 0 to 7 h. The measurement data are presented as the mean ± SD of two separate experiments conducted in duplicate (N = 4). * indicates (*p* < 0.05) compared to the control group. ** indicates (*p* < 0.01) compared to the control group. *** indicates (*p* < 0.005) compared to the control group.

The comparison between the different treatments revealed interesting findings regarding their effects on cell migration and wound closure. Treatment with the vehicle and non-formulated CPB led to an increase in cell migration and demonstrated a similar pattern of wound closure. However, treatment with formulated probiotics resulted in a greater enhancement of wound closure, indicating a synergistic effect between the CPB and the vehicle. The statistical analysis of the AUC of wound closure, depicted in Figure 8, demonstrated a noteworthy enhancement in all treatment groups when compared to the untreated control. However, there were no significant variances observed among the three treatments. Nevertheless, the formulated probiotic displayed a greater increase in AUC of wound closure (20%) compared to the non-formulated probiotic (16%) and the vehicle (14%) when tested at the highest concentration of 750,000 CFU/mL.

## 3. Discussion

Wound healing is a network of interaction between skin cells, the immune system, and external factors including microorganisms and chemicals. This process can be disrupted by an imbalanced skin microbiota. Ming et al. and Lizardo et al. found that probiotics of genus Lactobacillus protected the wound from pathogenic bacteria and had anti-inflammatory capabilities [4,12]. In another study, *Staphylococcus epidermidis*, a skin commensal bacterium, was shown to produce anti-inflammatory lipoteichoic acid [13]. The use of probiotics in wound healing is poorly described, especially in animals. Only a few articles describe the potential use of probiotics in pet care [14]. In this study, we investigated the implication of probiotics in wound healing in dog keratinocytes in vitro to provide support for the development of a probiotic-based topical cocktail intended for canine care purposes. The product was tested using a newly established in vitro skin model using CPEK. The canine keratinocytes were used to reconstruct a monolayer of cells with simulated wound scratch [15]. This assay allowed for the assessment of active ingredients within the PPP and to evaluate the implication of the CPB in wound closure. To our knowledge, this study represents the first utilization of the in vitro scratch test on canine keratinocytes to test probiotics’ effect on wound healing. While challenges were encountered due to the differences between human and dog keratinocytes, such as the ineffectiveness of TGF-beta in promoting wound healing in dog keratinocytes (internal data), these findings underscore the necessity of conducting species-specific investigations and studying wound healing mechanisms directly in canines. By addressing these gaps in knowledge, this research contributes valuable insights to the potential of probiotics for promoting wound healing in dogs. The CPEK were only used by Gagnon et al. (2016) and Lertpatipanpong et al. (2023) in the assessment of the low-level laser therapy and the cold atmospheric microwave plasma in wound healing in the canine skin model, respectively [16,17].

In our developed model, the viability of CPB in culture conditions was evaluated and indicated that concentrations higher than 75 CFU/mL led to better survival while lower concentrations led to challenging bacterial survival. In fact, CPEK stimulated with alive CPB potentially produce antimicrobial peptides (AMPs), e.g., defensins, as a first-line protection [18,19]. However, when the CPB concentration was increased, the resistance of probiotics was increased. The probiotics could produce AMPs proteolytic enzymes, bind and neutralize AMPs, or change the charge of their membrane to be less attracted by the AMPs [18].

Moreover, in the co-culture with PCB, the CPEK increased the expression of pro-inflammatory cytokines interleukin-8 (IL-8), IL-6, and tumor necrosis factor (TNF-α). IL-6 plays a central role in acute inflammation and is necessary for the timely resolution of wound healing [20,21]. Released early in response to injury, IL-6 induces the release of proinflammatory cytokines from tissue resident macrophages, keratinocytes, endothelial cells, and stromal cells. Both IL-6 and TNF-α stimulate keratinocyte proliferation and participate in the inflammation phase of wound healing through activating downstream cascades of immune response [22]. The IL-8 is a chemokine produced by macrophages, keratinocytes, and other cells. It mediates immune response in case of injury and infection. In dogs with cutaneous wounds, Avazi et al. (2019) found that IL-8 concentration in the serum of the injured dogs was double of the group control [23]. In an in vitro assay, Chermprapai et al. (2018) observed that when a canine keratinocyte cell line was exposed to *Staphylococcus pseudintermedius*, the expression of the IL-8 mRNA was 4-fold higher than in the untreated control [19]. In addition to being an immune response activator, IL-8 plays an important role in wound healing. In human keratinocytes, the IL-8 improves cell migration in wound healing [24]. While our study focused on analyzing the expression of IL-8, IL-6, and TNF-α, a broader range of cytokines should be evaluated to provide a more comprehensive and detailed characterization of the immune modulatory effects of the probiotic-based cocktail treatment.

To assess the impact of probiotic bacteria on cell viability, CPEK were exposed to various concentrations of both formulated and non-formulated CPB. To mitigate any potential influence of bacteria on the MTT assay results, rigorous washing steps were implemented prior to conducting the assay. These steps aimed to eliminate any bacteria present in the culture medium. Although it is possible that trace amounts of bacteria might have remained on the cells, their effect on the MTT results can be considered negligible. The cell viability was minimally affected by both types of probiotics at lower concentrations, with a decrease observed only at the highest concentration. This observation was an indicator of the effectiveness and safety of the recommended amount of PPP to be applied to the skin. The PPP would have less toxicity than commonly used cytotoxic disinfectants for the keratinocytes at the recommended dose [25,26]. The assessment of the impact of the vehicle on cell viability was not explicitly conducted in our experiments. However, the viability of cells exposed to the formulated CPB in the vehicle was found to be not significantly lower, except at the highest concentration, compared to the non-formulated CPB. This observation suggests that the vehicle itself does not exert a significant effect on cell viability.

It is worth noting that bacteria of the genus Bacillus have never been used in topical preparation for wound healing in animals in general [9,27]. Only one study reported the use of *Bacillus subtilis* to treat open wounds in vivo in mice as a model [28]. Several examples of formulation for human applications were reported where species of Bacillus with other species helped to treat skin microbiota dysbiosis and wound healing. However, *Bacillus* spp. was not the most cited compared to *Lactobacillus* spp. [patent US 10695386B2, CN 111601583A, US 6723326B1, EPO 975227A1], [29,30].

Our preliminary findings on accelerating wound closure serve as a foundational basis for future research in this field. While the scratch wound healing assay provides valuable insights into basic cell migration properties, it is crucial to acknowledge that relying solely on this assay is insufficient to draw definitive conclusions regarding the wound healing promotion potential of the probiotic cocktail (CPB). Therefore, additional experiments are necessary to comprehensively evaluate and establish robust evidence for the efficacy of CPB in promoting wound healing.

Although our study primarily focused on assessing the impact of the probiotic cocktail on Canine Progenitor Epidermal Keratinocytes (CPEK), we recognize the significance of addressing the broader impact on living systems and considering other cell types and tissue responses. Wound healing is a complex biological process that involves interactions among various cell types, including fibroblasts, immune cells, endothelial cells, and keratinocytes. These cells collectively contribute to the intricate cascade of events required for successful wound closure and tissue repair. Hence, exploring the effects of the probiotic cocktail on these different cell types would provide valuable insights into its broader influence on wound healing.

To achieve a more comprehensive evaluation, future experiments can incorporate advanced wound healing models such as 3D or full-thickness dog skin models, which offer a realistic reconstruction of the skin and provide more reliable results compared to monolayer models [31]. These alternative models not only enhance the validity of our findings but also align with essential regulations and guidelines, such as Directive 2010/63/EU and REACh [32], which prioritize the well-being and ethical treatment of animals in scientific investigations. Moreover, assessing the tissue response to the probiotic treatment is vital to understanding its overall efficacy and safety as a non-medical intervention. Factors including inflammation, angiogenesis, extracellular matrix remodeling, and immune modulation play critical roles in the wound healing process and can be influenced by the probiotic cocktail.

Additionally, investigating the specific molecular mechanisms underlying the observed effects of CPB represents another avenue to confirm its impact on wound healing. By identifying the most effective bacterial strains and optimal dosages, we can develop innovative probiotic-based therapies tailored to enhance wound healing in canines and potentially benefit other animals as well.

In this context, the probiotic-based topical cocktail (PPP), as a non-medical solution, would first undergo validation for wound healing in vitro before being recommended by veterinarians for the cleaning of mild wounds in dogs. Gathering feedback from users would serve as persuasive evidence of the product’s effectiveness.

## 4. Materials and Methods

### 4.1. Probiotic Product

The CBP containing spores of *B. subtilis, B. amyloliquefaciens, B.megaterium, B. pumilus,* and *B. licheniformis* was added into a chemical matrix referred to as the vehicle. In this form, the bacteria preserved their viability in the vehicle. The vehicle included a mixture of water, preservative, and pH regulator to obtain an acid (pH range of 2.9–3.9) aqueous suspension of 7.5 × 10^7^ spores/mL. This suspension is a prototype probiotic product (PPP) with potential therapeutic applications. PPP is intended to promote wound healing by contributing to the maintenance of healthy skin microbiota, to the debridement of the wound through probiotics enzymatic action, and to enhance the immune response of skin cells. The product is designed as a spray to maximize exposure of living bacteria to the skin. As the wound-healing efficacy was tested in an in vitro cell culture modal, the instructions for use would be to first shave the hair around the wound, then to remove impurities with lukewarm water, and to dry with a soft cloth. Next, the wound is sprayed with PPP and a bandage is applied. This procedure of cleaning should be repeated daily.

### 4.2. Cell Line and Culture Conditions

The Canine Progenitors Epidermal Keratinocytes cell line (CPEK) was obtained from CellnTec (Bern, Switzerland) and received at passage 35. For the study, cells at passage 44 and 59 were used. The CPEK cells were cultured in the CnT-09 growth medium supplemented with L-glutamine and 10% of fetal calf serum (FCS) provided by CellnTec and incubated in a humidified cell incubator maintained at 37 °C with 5% CO_2_.

### 4.3. Viability Assay of Probiotics

The ability of CPB to survive in treatment conditions was assessed using the LIVE/DEAD™ BacLight™ Bacterial Viability Kit according to the manufacturer’s instructions. The CPEK cells were treated with non-formulated CPB at varying concentrations of 75, 750, or 7500 CFU/mL for 24 h. The analysis was conducted using an Eclipse 80i inverted microscope (manufactured by Nikon Instruments Inc., Melville, NY, USA). The live bacteria were observed utilizing the FITC filter cube, which displays green fluorescence, while the dead bacteria were visualized using the TxRed filter cube, which displays red fluorescence. Live/Dead quantification was performed using ImageJ software version 1.53t.

### 4.4. Viability Assay of CPEK Cells

The CPEK cells were cultured in a 96-well plate and treated with non-formulated CPB at different concentrations ranging from 75 CFU/mL to 75,000,000 CFU/mL for 24 h. To measure cell viability, the CellTiter 96^®^ Assay (MTT viability assay, Promega France, Charbonnières-les-Bains, France) was employed. This assay depends on the conversion of tetrazolium salt by the cells into a formazan product, which can be easily detected with a 96-well plate reader at 570 nm. After extensive washing of the cells to remove the bacteria, the assay was carried out by introducing 15 µL of the Dye Solution to each culture well and incubating for 4 h in a cell incubator. Then, 100 µL of the Solubilization/Stop Solution was added to each well and left for one hour. The absorbance was detected at 570 nm using a GloMax^®^ Explorer plate reader (Promega France, Charbonnières-les-Bains, France). The measurements were performed in duplicate (N = 2).

### 4.5. Measurement of Cytokine Expression

The CPEK cells were treated with non-formulated CPB at different concentrations ranging from 75 CFU/mL to 75,000 CFU/mL for 24 h. At the end of treatment period, mRNA expression of inflammatory markers, IL-8, IL-6, and TNF-α was measured using quantitative real-time RT-PCR.

Total RNA was isolated using ReliaPrep™ RNA Tissue miniprep System (Promega France) according to the instructions provided by the manufacturer. The concentration of total RNA in each sample was measured using Eppendorf UVette^®^ and Eppendorf BioPhotometer plus.

Total RNA (500 ng) was converted into cDNA using High Capacity RNA to cDNA Master Mix kit according to the instructions provided by the manufacturer (Applied Biosystems, Foster City, CA, USA).

Real-time PCR was performed on an ABI 7500 Real-Time PCR System (Applied Biosystems). Validated PCR primers and TaqMan MGB-FAM labelled probes (TaqMan^®^ Assay on Demand; Applied Biosystems) were used in the study. The references of the sequences used are as follows: Cf02624283_m1, Cf02628236_m1, and Cf02624153_m1 for CXCL8, TNF-α, and IL-6, respectively. The housekeeping gene GAPDH (Cf04419463_gH) was used as reference gene to normalize for the level of mRNA in the two different groups.

PCR amplifications were performed in a total volume of 25 μL using TaqMan^®^ Universal PCR Master Mix No Amperase^®^ UNG according to the manufacturer’s instructions (Applied Biosystems). Thermal cycling parameters were as follows: Polymerase activation (10 min, 95 °C) followed by 40 cycles of denaturation (15 s, 95 °C) and combined annealing/extension (1 min, 60 °C). Each PCR reaction was performed in triplicate. The PCR fluorescence data were analyzed with 7500 software (version 2.0.6, Applied Biosystems). The increase in the expression of a target gene was expressed as fold change and calculated as 2^−ΔΔCt^.

### 4.6. In Vitro Wound Healing Assay

#### 4.6.1. Scratch Test

To investigate the migration of the keratinocytes, an in vitro scratch test was performed. The Ibidi^®^ µ-dishes with silicone culture inserts were utilized, creating a cell-free pseudo-wound field with a well-defined width of 500 μm. The cells were then seeded into the two compartments and allowed to proliferate until confluency was reached. The culture medium was changed for a medium containing 1% FCS. Serum starvation lasted 5 h after that the insert was removed to create a precise and reproducible cell-free gap or wound.

Cells were subject to different treatments. The CPEK were exposed to formulated and non-formulated CPB (concentrations tested of CPB were 7500, 75,000, and 750,000 CFU/mL for both treatments), and to the vehicle used in the formulation of PPP (diluted to 0.031%, 0. 31%, 3.1% equivalent to the dilution of the formulated CPB). The untreated cells were used as a control, and cells treated with taxol (Biotechne, Minneapolis, MN USA) at 100 nM were used as a reference for the inhibition of cell growth. After treatment, cells were placed in a humidified cell incubator at 37 °C with a CO_2_ concentration of 5%. Each treatment condition was replicated using two separate biological replicates (N = 2), each experiment was repeated twice, and the entire experiment, including all treatment conditions and their replicates, was repeated twice.

#### 4.6.2. Live Cell Imaging and Analysis

Quantitative data on cell migration and gap closure rate were obtained using an automated image analysis platform. Photographic images were captured every 60 min over a 20 h period using a CytoSmart Omni brightfield device equipped with a high-resolution digital 6.4 MP CMOS camera. The Omni CytoSmart device operated in the CO_2_-incubator to ensure stable and controlled conditions during images acquisition.

The acquired images were automatically analyzed using the CytoSmart algorithm, which accurately identified and highlighted the scratch areas (open wound areas). To assess the quality of algorithm integration for gap detection, videos of the image acquisition process were generated. The analysis of the scratch images was conducted using the CytoSmart software AxIS Vue version 1.0.41346, which calculated the open wound area for each image in µm^2^/h, providing quantitative measurements of the extent of wound closure over time. Cell migration was analyzed for 7 h.

The percentage of the open wound area was calculated as follows:

$$\mathrm{Wound}\;\mathrm{area}\;=\frac{\mathrm{Wound}\;\mathrm{area}\;\left(t\right)}{\mathrm{Wound}\;\mathrm{area}\;\left(0\right)}\times 100\%$$

The percentage of wound closure was calculated from the percentage of the open wound area as follows:

Wound closure = 100 − Wound area (%)

The percentage of wound closure was plotted over time for each treatment condition. Data are presented as mean ± standard deviation (SD). The scratch area at time point 0 h was set to 100.

Additionally, for each treatment condition, the area under the curve (AUC) was calculated between 0 and 7 h using the formula:

$$\mathrm{AUC}\;=\;\sum_{t=0}^{7}\frac{\mathrm{Wound}\;\mathrm{closure}\;\left(\mathrm{tx}\right)\;-\;\mathrm{Wound}\;\mathrm{closure}\;\left(\mathrm{tx}+1\right)}{2\Delta t}$$

AUC values are expressed as AU*h, and data are presented as mean ± SD.

#### 4.6.3. Statistical Analysis

The wound healing experiments were performed in duplicate on two separate occasions, resulting in a total of four samples. Additionally, the viability assay was conducted once with duplicate samples, resulting in a total of two samples. We acknowledge that the number of experiments and samples used may be considered limited for a comprehensive statistical analysis. However, it is important to note that the inclusion of duplicate samples was intended to enhance the reliability of our findings. Considering the limitations in the number of experiments and samples, we recognize the need for caution in the interpretation of the statistical results. It is essential to understand that the statistical analysis provided is primarily for informational purposes, offering initial insights into the data.

The statistical analysis included the use of an F-test to assess the equality of variances, followed by a Student’s *t*-test to compare the means of different conditions. The null hypothesis, which states that the means are equal, was rejected at a significance level of *p* < 0.05.

## Data Availability

Not applicable.
